# Influence of context on quality improvement priorities: a qualitative study of three facility types in Lagos State, Nigeria

**DOI:** 10.1136/bmjoq-2021-001532

**Published:** 2022-03-28

**Authors:** Abimbola Ayodele Olaniran, Modupe Oludipe, Zelee Hill, Adedoyin Ogunyemi, Nasir Umar, Rebecca Ayorinde, Kelechi Ohiri, Joanna Schellenberg, Tanya Marchant

**Affiliations:** 1 London School of Hygiene and Tropical Medicine Faculty of Infectious and Tropical Diseases, London, UK; 2 Health Strategy and Delivery Foundation, Lagos, Nigeria; 3 Institute for Global Health, University College London, London, UK; 4 University of Lagos College of Medicine, Lagos, Nigeria

**Keywords:** quality improvement, health policy, maternal health services, qualitative research

## Abstract

**Background:**

Quality improvement (QI) collaboratives are increasingly popular. However, there is a need for an in-depth understanding of the influence of context on its implementation. We explored the influence of context on the change concepts considered by public primary (primary health centres), public secondary (public hospitals) and private (private facilities) collaboratives established to improve maternal and newborn health outcomes in Lagos State, Nigeria.

**Methods:**

Between February 2019 and January 2020, we conducted a qualitative study using meeting reports, key informant interviews and participant observation. Data were analysed using the high-quality health system framework for assessing health system and user experience that distinguished three quality domains: quality impacts, processes of care and health system foundations.

**Results:**

Nineteen change concepts and 158 change ideas were observed across 28 facility QI teams. Change concepts and ideas prioritised were influenced by government and non-governmental leaders but ultimately shaped by facility QI capacity, time allocated for QI activities and availability of local data. Of the three quality domains, process of care, including patient satisfaction, received the most attention across facility types. There was considerable variation in the change concepts considered across domains. For example, more public hospitals focused on complication management because of a relatively high prevalence of and capacity to manage maternal complications; primary health centres focused more on complication referrals, while private facilities prioritised revenue generation. Problems with availability of resources were particularly highlighted in primary health centres which had relatively less financial commitment from stakeholders.

**Conclusion:**

Our findings provide insights into QI collaboratives’ mechanism of change in which external stakeholders, including government, drove QI priorities for action but the ultimate decisions depended on local realities of facilities. Our findings underscore the need for strong QI leadership and sufficient resources to enable facility QI teams to prioritise change concepts for greater health impact.

**Key messages**

**What is known about the subject?**

- Contextual factors are known to affect the implementation of facility-level quality improvement (QI) but there is a lack of evidence about how context influences the prioritisation of change concepts when QI is implemented in low-resource settings.

**What this study adds?**

- Working in primary healthcare facilities, hospitals and private hospitals engaged in QI in Lagos State, Nigeria, this study describes an inductive approach to mapping change ideas using a quality framework.

**How this study might affect research, practice or policy?**

- Understanding the internal and external contextual factors that influence priorities in different facility levels could be leveraged to support implementation of QI initiatives in low-resource settings.

The study provides new insight into the mechanism of change of quality improvement (QI) at facility level, observing that QI priorities are shaped by both internal and external contextual factors, with the ultimate decision for QI action in facilities depending on internal factors.

## Background

Improving the quality of care provided in health facilities is essential to achieve Sustainable Development Goal 3 on health.^1 2^ One of the key strategies is QI collaboratives using the breakthrough collaborative approach^3 4^ which seeks to achieve large-scale improvements by bringing QI teams from different health facilities from similar settings together to improve processes in their respective facilities with the support of QI subject experts. These teams are expected to apply QI learning to their facility contexts by developing, testing and implementing change ideas that could address a change concept.

Change concepts encompass problems that teams want to address, grouping actionable ideas for changing a process.^5 6^ Given the emphasis on local problem solving, it is important to acknowledge the influence of context on the change concepts prioritised by QI teams.

For this study, we defined contextual factors as a set of characteristics and circumstances internal to the organisation (eg, local priorities of a facility type) and those external to the organisation including (eg, leadership and governance of the state health system) that interacts, influences, modifies, facilitates or constrains an intervention and its implementation.^7–9^

Understanding how context influences QI priorities could provide insight into the mechanism of change, and this could be leveraged to shape implementation for maximum benefit.^8 10 11^ This is important in Lagos State, Nigeria, where the maternal mortality rate (MMR) was estimated at 450 deaths per 100 000 live births in 2008 and neonatal mortality rate (NMR) of 29 deaths per 1000 live births in 2016^12 13^: both estimates being far higher than the Sustainable Development Goal country targets of MMR less than 140/100 000 and NMR less than 12/1000 by 2030.^1 14^ Seeking to improve maternal and newborn outcomes and patient satisfaction, the Lagos State Ministry of Health, the Primary Health Care Board and managers of private facilities implemented the Nigeria Healthcare Quality Initiative (NHQI), a QI intervention using a modified collaborative learning approach.^15^ An initial pilot phase ran from November 2014 to September 2017, followed by a scale-up phase between November 2017 and October 2020. Three facility types were enrolled: public primary healthcare centres (PHCs), public secondary hospitals and private facilities. The modified collaborative learning approach entailed capacity building through three collaborative learning sessions (one for each facility type) and more local cluster meetings to bring QI teams together for peer-to-peer learning. The three collaboratives had a shared goal to reduce facility-based maternal and neonatal mortality and improve patient experience through broad change concepts, but it was anticipated that their complex and diverse needs, priorities and interests would result in numerous local adaptations to those change concepts.^16^

This study sought to understand whether and how the change concepts differed across facilities in Lagos State and examined how differences were influenced by contextual factors relating to facility type, health system and the stakeholders.

## Methods

### Study design

We conducted a qualitative multiple-case study in which we defined a case as a collaborative of each facility type.

### Study setting

The study took place in Lagos State, Nigeria with a population of about 24 million people and over 10 000 skilled health workers providing services across 3 tertiary hospitals, 26 public secondary hospitals, 333 PHCs and 2886 private facilities.^17^ About 27% of deliveries occur in public facilities, 48% in private facilities and 25% at home or other locations.^18^

Lagos State Government works with organisations to implement quality initiatives in facilities (table 1), ensures patient rights are protected through its service charter initiative^19 20^ and leverages the State Health Insurance Scheme to achieve universal health coverage.

**Table 1:** Organisations implementing quality initiatives in Lagos State

Organisation		Role description
Government	Health Service Commission	Governs and supports state-owned secondary health facilities.^26^ Staffs the public secondary hospitals and by extension, regulates their practices. Joint facilitation of collaborative learning sessions and cluster meetings.^15^
	Hospital Management Board	Facility-level committee comprises the hospital’s medical director and heads of departments/units of state-owned secondary health facilities responsible for the day-to-day running of each hospital.^26^
	State Primary Healthcare Board	Oversees activities of all the PHCs.^15 26^ Joint facilitation of collaborative learning sessions and cluster meetings.^15^
	Local Government Area Council	Finances the day-to-day running of the PHCs located within its area council, including funding implementation of change ideas.^15 26^
Non-governmental	Health Strategy and Delivery Foundation	Coimplemented NHQI with the state government. The NHQI focused on building the technical capacity of health workers on the use of QI methodologies to improve health processes and outcomes. The initiative entailed establishing collaborative learning sessions, coaching and mentoring, and supporting the facilities to conduct QI team meetings to develop change ideas that are expected to lead to an improvement.^15 26^ Joint facilitation of collaborative learning sessions and cluster meetings.^15 26^
	PharmAccess foundation	Implements the SafeCare initiative that ranks the various facility types based on international standards and processes, thereby serving as a stimulus for providers to improve the quality of healthcare delivery.^27^
	Evidence-4-Action-MamaYe	Supports the state ministry of health and public hospitals in implementing Maternal and Perinatal Death Surveillance and Response (MPDSR) by measuring the prevalence of maternal and perinatal deaths, identifying the root causes of these deaths and proffering solutions.^28^
	Saving One Million Lives	Seeks to improve access to essential primary healthcare services for women and children by driving institutional processes to improve health outcomes.^29^

NHQI, Nigeria Healthcare Quality Initiative; PHC, primary healthcare centre; QI, quality improvement.

### Study population

The eligible study population included all 50 facilities (6 PHCs, 19 public hospitals and 25 private facilities) enrolled in the NHQI scale-up phase. These were facilities with: (i) perceived will and commitment of leadership to engage in QI activities; (ii) high volume of maternal and neonatal cases; (iii) sufficient staff numbers to enable the formation of a QI team within the facility and (iv) availability of a data manager to organise and make facility health data accessible to the QI team.

### Data collection

Between February 2019 and January 2020 we reviewed QI team meeting reports, conducted key informant interviews with state and facility stakeholders and observed collaborative learning sessions and cluster meetings (table 2).

**Table 2:** Data collection

Study objective	Data collection method	Data source
To document change concepts considered by each of the three facility types	Meeting reports	QI team meeting reports
To understand how change concepts considered were influenced by the context of the three facility types, the Lagos health system and the stakeholders	Key informant interviews	State and facility-level actors
	Participant observation	Collaborative learning sessions, cluster meetings

1. Meeting reports: we approached all 50 facilities to access reports of the QI team meetings between February 2019 and January 2020. The report template comprised sections on the problem description, method of problem identification, aim statement, change ideas and measures used to track performance.
2. Key informant interviews were conducted in English, with participants purposively drawn from state-level government agencies, non-governmental organisations (NGOs) involved in QI, and from enrolled facilities.

An initial list of government agencies and NGOs for interview was identified from discussions with the NHQI implementer and the list grew to include other organisations based on emerging findings.

State-level participants (state government and NGO-level stakeholders) were eligible if they played an active role in NHQI design or implementation or were involved in other projects with possible facility-level interactions with NHQI. Facility-level participants were selected from the facilities that regularly submitted QI team meeting reports. QI team members who regularly attended facility QI team meetings, were knowledgeable about the QI activities in the facility, and who consented to a recorded interview were selected.

Potential participants were initially contacted over the phone or by email, an information sheet provided, and the content discussed before asking for written consent for a face-to-face interview. We used a topic guide to ask state and facility level participants about the operationalisation of QI at the state and facility levels, including what and how change concepts were considered. Data were collected until saturation was reached when additional data did not provide new information.^21^

1. Non-participatory observations were conducted at collaborative learning sessions and cluster meetings for each facility type, identified through opportunistic sampling: the researcher (AO) attended all the meetings he was aware of.^22^ For each meeting, the participants were notified that the session was being observed while the researcher made notes.

### Data analysis

Data analysis was guided by the high-quality health system framework for assessing health system and user experience.^2^ The framework describes three domains (quality impacts, processes of care and health system foundations) and 10 subdomains (table 3 in the Results section). QI meeting reports (labelled according to facility type) were reviewed for information on change ideas. Using an Excel template, these change ideas were inductively organised into broader change concepts before mapping against the 10 subdomains of the quality framework.

**Table 3:** Summary of change concepts, problems, and change ideas developed by facility type

Domain	Quality subdomain	Change concept	No of distinct problems described	No of change ideas tested	No of facilities that tested the change ideas for this concept		
					PHC (N=6)	Public hospital (N=14)	Private facilities (N=8)
Quality impact	Better health	Strengthen complication management Strengthen complication identification	7	31	4	12	4
	Confidence in system	–	0	0	0	0	0
	Economic benefits	Increase revenue generation Ensure patient financial protection	6	12	0	0	2
Process of care	Competent care and system	Ensure disease prevention & health promotion Improve documentation Improve service uptake and continuity	21	40	3	7	6
	Positive user experience	Reduce waiting time Improve ease of accessing care Protect patients’ dignity Strengthen staff–patient relationship Ensure clean and conducive environment Provide quality meals	17	44	5	10	4
Foundations	Population	–	0	0	0	0	0
	Governance	Ensure political buy-in of the management	1	1	0	1	0
	Platform	–	0	0	0	0	0
	Workforce	Improve staff welfare Ensure staff discipline	8	11	1	2	3
	Tools	Improve availability of commodities Improve availability of equipment Ensure supply of utilities, for example, water	10	19	4	6	2
Total		19	70	158			

PHC, primary healthcare centre.

A coding template was subsequently developed in the NVivo V.11 software,^23^ which was used to sort interview data. Transcripts were labelled and read several times to familiarise and understand the various components of contexts that informed the considerations given to certain change concepts.

Constructs relating to context such as leadership and prior QI team experience were identified from QI literature.^7 8^ These constructs provided a lens for reviewing the transcripts for characteristics that may influence priorities, such as facility’s level of care, QI team competence, needs and priority; and availability of leadership, political and financial support at state and facilty levels. Subsequently, the influence of these characteristics on decision making around change concepts was explored.

The observations from collaborative sessions were used to (in) validate findings from facility QI team meeting reports and transcripts which largely reflect findings from facilities with active QI teams. Where necessary, findings from observations also informed iterative revision of the topic guide to facilitate exploration of new emergent sub-themes.

Two researchers (AO, TM) reviewed the codes for validity to ensure that they accurately reflected the subdomains and represented the data. These researchers regularly discussed to aid conceptual thinking and to increase analytic rigour. Additionally, analysis workshops were held with a larger team of implementers and researchers during and after the period of data collection. Data validity was ensured by triangulating findings from the QI team meeting reports and interview transcripts with notes written during observation of learning sessions and cluster meetings for each facility type. Reflective notes were also kept throughout data collection and analysis.

### Patient and public involvement

Patients and/or the public were not involved in the design, or conduct, or reporting, or dissemination plans of this research.

## Results

### Data summary

From February 2019 to January 2020, a total of 140 QI team meeting reports were submitted by 28 of the 50 NHQI facilities (6 PHCs, 14 public hospitals and 8 private facilities). Forty-five key informant interviews were conducted, and 17 learning sessions and cluster meetings observed (table 4). Except for one public hospital, all facilities that participated in interviews submitted QI team meeting reports. Three of the seven governmental and NGOs invited for key informant interviews declined participation.

**Table 4:** Overview of data collection*

Data collection method	State (government and non-governmental)	PHC	Public hospital	Private facility	Total
Meeting reports		15 QI meeting reports (from 6 PHCs)	87 QI meeting reports (from 14 hospitals)	38 QI meeting reports (from 8 private facilities)	140 reports from 28 facilities
Key informant interview	12 (in 4 organisations)	9 (in 3 PHCs)	11 (in 4 hospitals)	13 (in 7 private facilities)	45 interviews from employees of 14 facilities
Observation of meetings		5 cluster meetings	2 learning sessions 6 cluster meetings	2 learning sessions 2 QI leadership training	17 observations

*To protect anonymity, a detailed breakdown of interviewees is not provided.

PHC, primary healthcare centre; QI, quality improvement.

### Domains of QI

Table 3 draws on the QI team meeting reports to map change concepts, problems and change ideas across the three domains of the framework. A total of 158 change ideas were developed to address 70 identified problems that are grouped under 19 different change concepts, all of which were defined locally to contribute to the shared goal of improving health outcomes and patient experience. Compared with other domains, processes of care had more change concepts considered and problems addressed through change ideas. A complete list of problem descriptions and change ideas developed is included in the online supplemental annex 1.

10.1136/bmjoq-2021-001532.supp1 Supplementary data

### Quality impact

Quality impact includes three subdomains. While all facility types worked on better health outcomes, only private facilities had a change concept for economic benefits. No facility worked towards increasing confidence in the system.

#### Better health outcomes

All three facility types prioritised better health outcomes (4/6 PHCs, 12/14 public hospitals and 4/8 private facilities) although their change concepts varied. For example, management of complications was most popular in public hospitals which had a relatively high case prevalence of maternal complications that might lead to deaths, such as eclampsia. Explaining why this was less popular in private facilities, a state-level participant mentioned:

So, some private facilities did it [used checklist on adherence to eclampsia protocol] but we [QI leadership from government and non-governmental organizations] didn’t roll it out collaboratively because private facility mothers were not dying … So, we can’t roll out eclampsia checklist across the whole, but because it wasn’t a problem for them, data didn’t show that it was a problem for them.

With state-level support, public hospitals were also more likely to have a Maternal and Perinatal Death Surveillance and Response committee that examined the causes of mortality. A state-level participant explained that

There is a committee for MPDSR [Maternal and Perinatal Death Surveillance and Response] in every [public] hospital … facility uses data from MPDSR file to form quality improvement project [develop change ideas]. For instance, if they had a death and the data showed there was a quality gap, the quality improvement team can work on it.

Another state-level participant highlighted the external influence in the form of supervisory support by one of the NGOs,

We [QI leadership from government and non-governmental organizations] also support the facility MPDSR committees with supportive supervisory visits, … visit these facilities when they are holding their committee meetings to actually monitor the process and see how we can provide one support or the other … We also hold a stakeholder meeting where we bring all the medical directors of these facilities and the officers both perinatal and maternal with the stakeholders, policy stakeholders to look at what the issues are … action plan is also developed ….

No PHC considered management of complications, consistent with the protocol to refer women with complications. Instead they focused on identifying and referring complications, as explained by a state-level participant,

‘Things like if they see a woman with high blood pressure they will refer, so you can’t be checking complication management, do you get?’.

Another limiting factor was thought to be that PHCs tended to employ non-specialist doctors who were less able to make root cause analysis of obstetric or paediatric complications. As explained by a state-level participant,

‘… also because of manpower. So we were able to form more robust teams in the GHs [public hospitals], but what you have in the primary healthcare centres you just have one, you have doctors but the doctors are not that involved in MNCH [Maternal, Neonatal and Child Health], they were involved in GOPD [General Outpatient Department].

#### Economic benefits

During this study period, only private facilities (2/8) prioritised the economic impact of healthcare provision to the facility and patients; Inefficient patient billing systems, inadequate stock-taking and cost-cutting through employment of individuals on national assignments with requisite skills but lower salary requirement were listed. This focus reflected the priority of private facilities to ensure financial security before investing in QI infrastructures. In the words of a state-level participant,

‘*…* financial bits affect QI because some MDs [private facility medical directors] will tell you, QI is taking away money from my hospital because you need to do somethings right. … So, what we are looking at is: can you get your financial structure right?’.

One private facility considered a change concept on patient financial protection, seeking to manage patient costs by ensuring that only essential investigations were requested.

### Processes of care

All facility types prioritised the two subdomains of the processes of care: competent care and systems and build positive user experience.

#### Competent care and systems

PHCs (3/6), public hospitals (7/14) and private facilities (6/8) prioritised competent care and systems, grouped into three change concepts: disease prevention and health promotion; documentation to aid diagnosis, treatment and decision-making; and improving service uptake through awareness creation and tracking continuity of care.

These concepts were largely externally motivated by the targets set by Lagos State Ministry of Health targets and partner NGOs. Explaining this, a state-level participant mentioned how satisfactory performance was important for accreditation and enrolment of facilities into the Lagos State Health Insurance Scheme,

‘… in preparation for the Lagos State Health Insurance Scheme, … the [non-NHQI partner’s] assessment is quite important to the state, every facility wanted to do well’.

Furthermore, the participant added that sometimes facility QI teams had to balance the demands of several QI partners to manage their workloads,

… they [a non-NHQI initiative] used the same quality improvement team that was set up for NHQI activities. So I think the burden of the work was a lot on the quality improvement team so at some point they [QI team] had to decide, do we come up with change ideas, or do we just work with the quality improvement plan of [the non-NHQI initiative].

Corroborating the importance of the NGO’s assessment of facilities, a public hospital QI team member said,

‘… we were doing Lagos State mandate and Lagos State was backing us, they came with the NGO assessment of facilities. Part of the motivation was seeing that you were having results … and the fact that Lagos State was scoring [assessing facility performance]’.

#### Creating positive user experience

This subdomain was consistent with the state’s service delivery priority to improve patient experience and satisfaction and was addressed by PHCs (5/6), public hospitals (10/14) and private facilities (4/8). A state-level participant explained,

Last year, we [QI leadership from the government] were able to come together as the PHC board and as the State Ministry of Health to come up with a quality policy … the State is moving towards improvement of patient’s experience, patient satisfaction, …improvement of service delivery as a whole. The other policy … is the service charter and the concept of the service charter is very similar to what they have in QI. … It’s a cross-cutting state implementation for improving client satisfaction.

Nonetheless, each facility considered local realities and problems based on data when considering change concepts. For example, to reduce waiting times, facilities considered punctuality of health providers, staggering patient appointments, having additional service units and making patient navigation easier … A PHC QI team member explained,

we have space constraints, … we have challenges with the flow, there is a way the structure is, we just have to look for a way that will work for us, if you go to the consulting room, you just have to walk back to the lab and back to the consulting room, it is not really a smooth flow, it is not purpose-built.

Six public hospitals and four private facilities prioritised ease of accessing services, patient dignity and strengthening staff–patient relationships. The importance of respectful communication was being reinforced through multiple channels in the state, including from state government health teams. A public hospital QI team member explained,

‘… we have representatives from HSC [Health Service Commission of the Lagos State Ministry of Health] coming to discuss with us and telling us how our attitude affects our treatment outcomes, how it can lead to limitations if care is not taken …’.

Patient feedback motivated two public hospitals to consider change concepts on providing quality meals. A state-level participant, however, said that prioritising ‘easier tasks’ could reflect the limited QI capacity of new QI teams,

it’s easier to work on kitchen than to work on mortality … the first time the facilities were presenting change ideas … they will work on the easiest things to tackle. I think mid-way into cluster meeting [mid-way into NHQI project], we started demanding that they come with their data. We were able to tell them that how come you are working on kitchen when people are dying …

Second, with limited time allocated to undertake QI activities it was difficult to engage with more complex problems. The state-level participant added

‘we noticed that if you leave the facility, they will work on easier things … they have their primary responsibility, and they do that full time. So, QI is an ad-hoc for them’.

### Foundations

Governance, workforce and tools were prioritised by all facility types, although with varied frequency.

#### Governance

A public hospital QI team emphasised the role of facility management in change uptake,

… there can never be QI team without the management. If you come with an idea and the management is not in support, there is no way it can be implemented. … because without the support, we can’t penetrate those departments. Once they know that the MD knows of what is happening, they [facility staff] won’t have a choice than to key in, …

#### Workforce

Improving staff welfare and ensuring staff discipline were considered by PHC (1/6), public hospitals (2/14) and private facilities (3/8). Public hospitals and private facilities addressed staff welfare by incentivising staff and promoting staff rights. A private facility QI team member explained,

Hmm when I mean staff welfare … I’m talking about the structure, the structure is not the building, we are talking about the people … the things that make people want to do more, they are being appreciated, they are being motivated, they are being encouraged to put in their best.

A PHC and the three private facilities considered the importance of ensuring staff discipline through processes and structures that encourage punctuality and formal dressing.

#### Tools

Irrespective of type, facilities prioritised availability of tools (PHC (4/6), public hospitals (6/14) and private facilities (2/8)) but the change concepts differed, reflecting the varied access to tools, utilities and commodities. Availability of commodities was considered by five public hospitals (eg, blood to manage postpartum haemorrhage) and one private facility. A recent health system policy banning mandatory blood donation by husbands of pregnant women seeking to register pregnancy resulted in limited availability of blood, leading public hospitals to promote and incentivise voluntary blood donation within the hospital and host community.

Change concepts on ensuring adequate supply of utilities such as power and water were limited to three PHCs, reflecting their reliance on the local government council’s political and financial commitment, but this was often lacking.

Explaining this challenge, a PHC QI team member stated,

‘Again in the family planning, the water is not running, the toilets are not functioning, the sink is not working … I can bet it when we tell the chairman [local government chairperson], we can be on it for a year. He will say write book [a lot of documentation]’.

Consequently, the PHCs resort to advocating organisations outside the local government council for support including provision of tools and basic amenities. Another PHC QI team member explained,

‘if you talk about challenges, what could it be? it has to be financial, … maybe some organizations can assist as part of their CSR [corporate social responsibility]’.

## Discussion

During a 12-month period, 28 of the 50 NHQI facilities submitted documentation showcasing the change concepts and change ideas they worked on to reduce facility-based maternal and neonatal mortality and improve patient experience. A total of 19 change concepts and 158 change ideas relevant to 70 distinct problems were extracted from these reports. Our findings reveal that some QI priorities were common across facility types, often driven by the health system leadership and external stakeholders, including government or NGOs. But many priorities were shaped by facility-level context such as availability of clinical subject experts, available time and capacity of the facility QI teams, facility culture, magnitude of a problem according to data, the level of care expected of a given facility type and available finances.

The public hospitals and, to some extent, private facilities, but not PHCs, focused on complication management to enhance better health outcomes. This focus was explained by the level of care expected of public hospitals, a relatively high prevalence of maternal complications, availability of external support to conduct maternal death reviews, and availability of specialist doctors. Conversely, change concepts relating to tools, including utilities such as water and power supply, were mainly present in PHCs. Evidence on effective QI implementation across income settings suggests that availability of tools are essentials for an enabling environment for the workforce.^3 7^ Accordingly, an inadequate implementing environment may have limited the QI activities of PHCs, obliging them to prioritise utilities before considering change ideas that directly addressed patient care.

Individual leaders from government and NGOs play critical roles in influencing QI priorities at the facility level^2 3 7 24^ through coordination, support, mentorship and coaching to strengthen capacity of facility QI team and staff.^3 24 25^ These leaders, or sometimes the lack of them, were found to be important in Lagos State where facility teams with least QI capacity prioritised many easy tasks, such as quality of meals, while failing to address problems that could have greater impact on health outcomes.^15^ Overall, we observed that the modified collaborative approach of NHQI^15^ resulted in considerable differences in the actions taken by facility QI teams, driven to a large extent by diversity of facility problems, priorities and exacerbated where leadership was lacking.

### Study strengths and limitations

This study makes key contributions to the body of knowledge on context and its role in influencing QI priorities across facility types within the same setting. Nonetheless, there are study limitations that need to be considered. The data reflects a snapshot of QI activities in only 28 of the 50 facilities enrolled in Lagos over 1 year: it was not possible to map information from facilities that did not submit reports. The mapping of change concepts, problems and change ideas was done together with key stakeholders in Lagos but was subjective and we did not study the extent to which change ideas were implemented. Finally, as in other qualitative studies, findings may not be generalisable beyond the study area, although many of the key findings appear to be consistent with literature from other settings.

## Conclusion

This study provides insight into the mechanism of change of flexible (modified) QI collaboratives in which external stakeholders including the government drove QI priorities while the ultimate decisions for QI action depended on the local realities of facilities. The importance of ensuring adequate facility capacity and QI leadership emerged as crucial implementation inputs in this real-world example of QI in Lagos State.

## Data Availability

Data are available upon reasonable request. The data are available from the principal investigator of the IDEAS project and coauthor for this manuscript, TM ORCID id 0000-0002-4228-4334. Reuse permitted on request.
