# Supplementary material for: Influence of context on quality improvement priorities: a qualitative study of three facility types in Lagos State, Nigeria
Source: BMJ Open Qual. 2022 Mar 28;11(1):e001532. doi: 10.1136/bmjoq-2021-001532 (PMC8961137; doi:10.1136/bmjoq-2021-001532)
Supplement: Supplementary data [file bmjoq-2021-001532supp001.pdf]

Annex: Map of change ideas and concepts across 3 quality domains, 7 sub-domains

Contents

1. Quality Impact-better health: 7 problems identified and 30 change ideas tested 2
2. Quality impact-economic benefit: 6 problems identified, 12 change ideas tested 4
3. Process of care-competent care and systems: 21 problems identified and 40 change ideas tested 5
4. Process of care-positive user experience: 17 problems identified and 44 change ideas tested 8
5. Foundations- governance: 1 problem identified and 1 change idea tested 11
6. Foundations- workforce: 8 problems identified and 11 change ideas tested 12
7. Foundations- tools: 10 problems identified and 19 change ideas tested 14

Olaniran AA, *et al. BMJ Open Qual* 2022; 11:e001532. doi: 10.1136/bmjoq-2021-001532

1. Quality Impact-better health: 7 problems identified and 30 change ideas tested

Under this domain, number of facilities documented to have identified problems and developed change concepts and ideas during the study period:

- 4 of 6 PHCs (labelled A1, A2, A3, A6)
- 12 of 14 public hospitals (labelled B2, B3, B5, B7, B8, B9, B10, B11, B12, B13, B17, B18)
- 4 of 8 private facilities (labelled C11, C15, C17, C20)

S/N	Problem description	Method of problem identification	Change ideas-*change concepts*
1	Health workers were not adhering to pre-eclampsia/eclampsia protocol; there was a delay in identifying and referring cases	- Facility register/case notes (B2, B5, B7, B9, B10, B12, B18).<br>- Findings from Quality Assurance Exercise (B2, B12, B13),<br>- Staff observation (B13<br>- MPDSR Fact Sheet (B12)<br>-Observation made by the ministry (B13)	Ensure checklist for tracking adherence is included as the 1st page of severe pre-eclampsia/ eclampsia patient case note-*complication management* (B2, B5, B7, B9, B10, B11, B12, B13, B17, B18).
			Sensitise doctors and nurses on the importance of using the checklist and adherence to protocol-*complication management* (B2, B5, B7, B9, B10, B11, B12, B13, B17, B18)
			Train/retrain doctors and nurses on the management of severe pre-eclampsia & eclampsia-*complication management* (B2, B13, B18).
			Develop and post pictorial protocol on the walls of the labour ward and share easily accessible protocol cards-*complication management* (B8, B12, B10); (C15)
			Appoint severe pre-eclampsia/ eclampsia champions who will ensure checklists are used-*complication management* (B18).
			Establish a triage protocol to facilitate prompt treatment and early referral-*complication management* (B5) (C11)
2	Health workers were not adhering to the protocol for managing post-partum haemorrhage (PPH); there was a delay in commencing appropriate care for patients with PPH	- From HSDF survey (B13)<br>- Facility register (B11)	Develop a checklist to monitor blood loss and signs of shock as per protocol-*complication management* (B12, B13)
			Sensitise labour ward & postnatal ward doctors and nurses on the importance of adhering to PPH protocol-*complication management* (B11, B12, B13).
			Senior doctors' daily ward rounds for prompt identification and management of PPH cases-*complication identification and management* (B11).
			Consultant OBGYN should be informed of all newly admitted women in labour-*complication identification and management* (B11).
			Train all clinical & non-clinical staff, including gatemen, on responding to emergencies such as PPH-*complication identification* (B11).
			Make close-user-group phone line avaialble to various service units e.g. laboratory, emergency room, wards etc. to facilitate within-facility communication-*complication management* (B11)
3	Increase in neonatal deaths from poor adherence to protocol on management of preterm babies; poor competence in neonatal resuscitation and delays in referral	- Facility register(B3),<br>- Review of case notes (B3)<br>- Staff observation (B5)	Include a management protocol & checklist in the case folder of preterm babies-*complication management* (B3)
			Train health providers regularly on management of preterm as per protocol-*complication management* (B3)

Olaniran AA, *et al. BMJ Open Qual* 2022; 11:e001532. doi: 10.1136/bmjoq-2021-001532

			Develop a protocol for referral of preterm deliveries-*complication management* (B5).
			Train doctors on neonatal resuscitation-*complication management* (B5, B8)
			Use a checklist to ensure that all steps in neonatal resuscitation are followed-*complication management* (B5).
			Educate health providers on the importance of early decisions and referrals-*complication management* (B5).
**4**	Partographs to identify labour complications are not properly filled.	Not documented	Inform the apex nurse to sensitise the labour ward nurses on the importance of filling the partograph properly-*complication identification* (A1, A2, A3)
			Identify a health provider to train/retrain nurses on partograph use-*complication identification* (C20)
**5**	Late identification of births at risk of birth asphyxia	Staff observation (B5)	WhatsApp group to educate pregnant women on signs of labour including danger signs-*complication identification* (B5).
			Ensure complete usage of partograph to guide decision making-*complication identification* (B5).
**6**	Increased maternal mortality and neonatal morbidity (e.g., birth asphyxia) from poorly managed complications referred from PHCs and TBAs	- Adopted from another facility (B8)<br>- Facility register (B11)	Visit/create an MPDSR WhatsApp group that includes health providers from the popular sources of referral to enhance communication, thereby enabling prompt referral-*complication management* (B8) (C17)
			Conduct MNH training of PHC staff and TBAs-*complication identification* (B8) (C17).
			Give monetary incentives to TBAs to encourage early referral of pregnant women-*complication identification* (C17)
			Encourage TBA-registered patients to come for ANC and delivery by promoting affordability through instalment payments-*complication identification* (C17)
			Educate pregnant women on the importance of delivery at PHC instead of using a TBA--*complication identification* (A6)
			Make hospital telephone line available to surrounding PHCs for prompt and seamless referrals-*complication management* (B11)
			Awareness about 24hrs and weekend services at the PHC to reduce TBA patronage-*complication identification* (A6)
**7**	More deaths at night/weekends from delay in identification and management of potential complications because fewer senior doctors, nurses and pharmacists were available then	Not documented	Increase the number of doctors and nurses on night shifts and weekend duties-*complication identification and management* (B8).

2. Quality impact-economic benefit: 6 problems identified, 12 change ideas tested

Under this domain, the number of facilities documented to have identified problems and developed change concepts and ideas during the study period:

- 0 of 6 PHCs
- 0 of 14 public hospitals
- 2 of 8 private facilities (labelled C17, C20)

S/N	Problem description	Method of problem identification	Change ideas – *change concepts*
**1**	Revenue generation affected by inefficient billing due to non-uniform price of services.	Not documented	Create a uniform price list and post it in doctor's office, ward and pharmacy to prevent underbilling-*revenue generation* (C20, C17)
**2**	Inadequate stock taking at the pharmacy causing a disparity in dispensation and stock and consequently unexplainable revenue loss	Routine data (C17)	Develop and implement a drug auditing policy- *revenue generation* (C17).
			Only drugs on prescription sheets should be dispensed- *revenue generation* (C17)
			Introduce stock-taking tools such as bin cards for monthly auditing of drug stock- *revenue generation* (C17)
			Access to pharmacy should be limited to pharmacy technicians- *revenue generation* (C17)
**3**	Patients were being admitted into the ward beyond their health insurance plan limit.	Not documented	Put a code on each admission slip to reflect the appropriate ward per health insurance plan- *revenue generation* (C20).
**4**	Staff members admission into VIP ward limited available bed spaces for patients who require admission	Not documented	Develop and read out admission policy during clinical/staff meeting- *revenue generation* (C20).
			Mandate doctors to inform their HODs about their intention to admit a member of staff or staff relative- *revenue generation* (C20).
			HODs of department to review admission plan of staff-- *revenue generation* (C20).
			Only one VIP ward should be assigned to staff members per time, and hierarchy should be considered- *revenue generation* (C20).
**5**	Inefficiencies due to staff shortage but the cost of employing new staff was too high.	Not documented	Employ trained individuals on national assignment (NYSC) who have requisite knowledge and skills, but salary may be less- *revenue generation* (C20).
**6**	Ordering of multiple investigations despite patients' financial constraints and health insurance limits.	Not documented	Senior doctors to review requested investigations and provide feedback to junior doctors on how to make judgement on the most important investigations-*patient financial protection* (C20).

Olaniran AA, *et al. BMJ Open Qual* 2022; 11:e001532. doi: 10.1136/bmjoq-2021-001532

3. Process of care-competent care and systems: 21 problems identified and 40 change ideas tested

Under this domain, number of facilities documented to have identified problems and developed change concepts and ideas during the study period:

- 3 of 6 PHCs (labelled A2, A4, A5)
- 7 of 14 public hospitals (labelled B2, B6, B7, B8, B9, B11, B15)
- 6 of 8 private facilities (labelled C5, C11, C16, C17, C20, C25)

S/N	Problem description	Method of problem identification	Change ideas - *change concepts*
**1**	Inappropriate umbilical cord care leading to increased risk of infection as some mothers lack understanding about the correct use of chlorhexidine gel	Not documented	Encourage and train mothers to use chlorhexidine gel for umbilical cord care- *disease prevention and health promotion* (C17)
			Include chlorhexidine gel as a delivery requirement - *disease prevention and health promotion* (C17)
			Solicit feedback from mothers on their experience with chlorhexidine through SMS, and WhatsApp platform - *disease prevention and health promotion* (C17)
			Liaise with apex nurse to facilitate training of nursing mothers on the use of chlorhexidine gel- *disease prevention and health promotion* (B8)
**2**	Some health workers do not understand the importance of disease prevention.	Not documented	Health talk on infection prevention and control including handwashing technique- *disease prevention and health promotion* (A4, A2)
			Train staff on the proper use of colour-coded bins, safety boxes and proper disposal of medical waste- *disease prevention and health promotion* (A2) (C5)
**3**	Staff members had needle stick injuries while attending to Hepatitis B positive patient, thereby needing hepatitis B immunoglobulin.	Staff observation (B6)	Deliver health talks to all staff on prevention of needle stick injuries and the need to get vaccinated- *disease prevention and health promotion* (B6)
			Screen staff for Hepatitis B- *disease prevention and health promotion* (B6)
			Procure and vaccinate Hepatitis B negative staff against the infection- *disease prevention and health promotion* (B6)
**4**	Increasing cases of hospital-acquired infection.	Not documented	Fumigate the ward at stipulated intervals- *disease prevention and health promotion* (B8) (C20, C5)

Olaniran AA, *et al. BMJ Open Qual* 2022; 11:e001532. doi: 10.1136/bmjoq-2021-001532

**5**	Hepatitis screening not routinely done during ANC preventing early detection of infection and increasing the risk of transmission to the foetus	Not documented	Develop and adopt a policy on routine hepatitis screening of women who visit the ANC and FP clinics- *disease prevention and health promotion* (C17).<br>Purchase screening kit and ensure continued availability - *disease prevention and health promotion* (C17)<br>Create awareness about hepatitis- *disease prevention and health promotion* (C17)<br>Referral of patients that test positive for hepatitis and track completion of referral- *disease prevention and health promotion* (C17)<br>Purchase screening kit and ensure availability- *disease prevention and health promotion* (C17)
**6**	HIV test not routinely done preventing early detection	Not documented	All patients presenting at the facility should be encouraged to undergo HIV counselling and testing - *disease prevention and health promotion* (C25)<br>Treatment/care/support should be provided for patients that test positive for HIV- *disease prevention and health promotion* (C25)
**7**	Poor knowledge of common health conditions among patients; few platforms for health education of patients	Not documented	Encourage health providers to educate patients on prevention of common ailments; provide information on the diagnosis and treatment of ailment they are being managed for; play documentaries on common health topics during peak hours-*disease prevention and health promotion* (B9) (C11, C5)<br>Post bills on notice boards and share pamphlets with health messages- *disease prevention and health promotion* (B7, B9) (C11)
**8**	Need to prevent unwanted pregnancies and abortions and improve uptake of services	Not documented	Conduct annual free/subsidised family planning outreaches to create awareness- *disease prevention and health promotion* (C17)
**9**	Need to create awareness on cervical cancer to improve uptake of services	Not documented	Conduct annual free/subsidised cervical screening- *disease prevention and health promotion* (C17)
**10**	Continuity of care is not ensured. Patient tracking system needs strengthening. Health outcomes not consistently documented because of the non-availability of referral register.	Not documented	Assign officer to track completion of referral over the phone – *service uptake and continuity* (A4).<br>Inform the M&E Officer to make requisition for referral register or improvise with hardcover notebook – *documentation* (A4).<br>Send reminder text messages to ANC clients before the date of next ANC visit– *service uptake and continuity* (B7) (C16).
**11**	Increase in under-5 pentavalent vaccine drop out.	Not documented	Identify and track defaulters (that had pentavalent 1 vaccine but missed an appointment for either pentavalent 2 or 3 vaccines) – *service uptake and continuity* (A2)
**12**	Need a system for timely action as patients requiring emergency care were sometimes kept in long queues because there is no triage nurse to sort patients based on case severity	Not documented	Advocate to MOH that a triage nurse be assigned to identify patients that would require emergency care– *service uptake and continuity* (A2)

Olaniran AA, *et al. BMJ Open Qual* 2022; 11:e001532. doi: 10.1136/bmjoq-2021-001532

**13**	Delay in transporting patients requiring emergency care from car park to emergency room thereby preventing timely action	Safe care recommendation (B15)	Provide an emergency ramp to transport patients requiring emergency care– *service uptake and continuity* (B15)
**14**	Decision making is affected by poor documentation leading to under-reporting, e.g., on use of chlorhexidine gel.	Not documented	Educate health providers on the importance of detailed documentation – *documentation* (A4, A5) (C16)
**15**	Lack of systematic assessment of patients for complications; conditions not appropriately assessed and managed	Not documented	Write high risk with red pen on the front cover of case folder - *documentation* (C20)
			Clinical audit of case notes especially patients at high risk of developing complications- *documentation* (C20)
**16**	Discharge summaries were not filled. During follow-up visits at the clinic, the doctor has to read through the entire or most part of the case notes to understand patient management during admission. This was affecting decision-making.	Staff observation (B11)	Sensitise the doctors including HODs on the importance of filling the discharge summary – *documentation* (B8, B11).
			Print more discharge summary forms and head of nurses in each ward should ensure that forms are available – *documentation* (B11).
			Appoint an officer/champion per ward to ensure summary sheet have been filled - *documentation* (B11).
**17**	Patients were not properly clerked	Not documented	Sensitise doctors on the need to clerk patients properly – *documentation* (B11)
			Develop and disseminate prototype of proper clerking among doctors – *documentation* (B11)
**18**	Blood samples were not properly labelled with a risk of mixing up blood samples and consequently results.	Not documented	A memo to be prepared and pasted in the injection room reminding the nurses to label every specimen bottle accordingly and send a notification to nurses on their WhatsApp platform – *documentation* (C20).
**19**	Medication errors from wrong prescriptions by doctors.	Not documented	Pharmacist should document and investigate medication errors and check with doctors to ensure wrong medications are not dispensed – *documentation* (C11)
**20**	Guidelines needed for management of health conditions	Safe care recommendations (B7)	Develop and disseminate protocols for management of pre-operative conditions – *documentation* (B7)
**21**	Need to ensure ease of use of emergency trolley during emergencies	Recommendation by the Lagos State Ministry of Health (B2)	Label content of the emergency trolley -*documentation* (B2)
			Develop a protocol to guide the use of emergency trolley – *documentation* (B2)

4. Process of care-positive user experience: 17 problems identified and 44 change ideas tested

Under this domain, number of facilities documented to have identified problems and developed change concepts and ideas during the study period:

- 5 of 6 PHCs (labelled A1, A2, A4, A5, A6)
- 10 of 14 public hospitals (labelled B2, B3, B6, B7, B8, B9, B11, B13, B15, B17)
- 4 of 8 private facilities (labelled C11, C17, C18, C20)

S/N	Problem description	Method of problem identification	Change ideas-*change concepts*
**1**	Long waiting time in the facility due to few service points that are far apart. May become worse during the rainy season as there are no roofed walkways between the buildings and people may have to enter the rain to move from one service point to another	Not documented	Relocate clinics to office spaces that are near laboratories and record office- *waiting time* (A1)
			Create multiple service point to cater for clinics and wards in different parts of the facility- *waiting time* (C20).
**2**	The patient spend time asking for direction to key service points as there are no signages.	Not documented	Develop signages to direct patients to key service points – *ease of accessing care* (A2).
**3**	Increased waiting time in laboratory due to only one investigation form was being used for haematology, microbiology & chemical pathology. Patients have to join long queues to photocopy and take a copy to each lab.	Not documented	Notify printer to print separate forms for each lab unit – *waiting time* (B6).
**4**	Patients are unsure about the cost of investigation and have to look for additional funds when money is insufficient.	Not documented	Develop and paste laboratory investigation price list – *ease of accessing care* (B6).
**5**	Single phlebotomy point with patients needing to wait in long queues to have their blood sample taken.	Not documented	Create multiple phlebotomy points with more than one phlebotomist at each point – *waiting time* & *ease of accessing care* (A2).
**6**	Patients were often anxious/agitated because they did not understand that some test such as MCS take 3 days to culture.	Not documented	Communicate average duration of the investigation to each patient- *ease of accessing care* (B6).
**7**	Investigation results had to be typed before being released.	Not documented	Doctors may request for the pre-typed (hand-written) form of the result if results are needed urgently – *waiting time* (B6)
			Deploy more admin staff to type and distribute investigation result – *waiting time* (B6).
**8**	Increased waiting time at medical records due to time spent retrieving case notes.	Not documented	Archive old case notes, (case notes of patients that are deceased or have stopped using the hospital – *waiting time* (B6)

Olaniran AA, *et al. BMJ Open Qual* 2022; 11:e001532. doi: 10.1136/bmjoq-2021-001532

			Institute electronic medical records – *waiting time* (A2) (B6)
**9**	Delay at the clinic due to inadequate number of doctors and nurses.	Patient complaints (B17) Complaint box (B17)	Reduce the number of doctors participating in ward round on clinic days to increase the number of doctors available for clinic consultation– *waiting time* (B17)
			Ward rounds should be of shorter duration on clinic days– *waiting time* (B17)
			Mandate doctors to commence consultation by 9.00 a.m. – *waiting time* (B17)
			Increase number of clinic days per week – *waiting time* & *ease of accessing care* (B17)
			Stagger patient clinic appointments to different times of the day and educate patients on need to comply with the appointments/send text to remind them of appointment – *waiting time* & *ease of accessing care* (A2, A6) (B13, B7)
			Task shift certain roles to CHEWs such as checking vital signs, administering injection and dressing wounds – *waiting time* (A4).
**10**	Patients go through a long process involving three visits before booking (register pregnancy) for ANC. The community members are not encouraged to attend the ANC clinics at the facility and may not come till delivery.	Facility register (B15)	Introduction of couples' clinic where couples are counselled on the importance of attending a recommended number of ANC clinics and husband is encouraged to donate blood voluntarily- *ease of accessing care* (B15)
			Health talks at out-patient clinics on the importance and process of registering pregnancy- *ease of accessing care* (B15) (C18)
**11**	Husband is expected to donate blood for ANC clients before registering pregnancy.	Facility register (B15)	Booking patients irrespective of whether they donated or not- *ease of accessing care* (B15).
**12**	Processes are expected to run sequentially such that one has to be completed before embarking on the next making it cumbersome	Staff observation (B11)	Allow the processes to run in parallel- *ease of accessing care* (B11).
**13**	Physicians were having to share a room for consultation, thereby compromising patient confidentiality & audiovisual privacy.	Safe care recommendation (B2)	Build additional consultation rooms for consultation- *patient dignity* (B2).
**14**	Need to improve patient involvement and participation in their health care delivery; strengthen the relationship with patients	Patient complaint (C17)	Develop a platform or leverage the QI committee to include one or more patients into the QI team or mini decision-making, e.g., patients-management forum- *patients' dignity and staff-patient relationship* (C17, C20).
			Hospital to employ a customer service agent to identify and address patient needs and expectations- *patients' dignity* (C20)
			Train staff on patient rights and customer relations- *patient dignity and staff-patient relationship* (B3, B15) (C17, C18)

			Birthday greetings to patients- *patients' dignity and staff-patient relationships* (C11)
**15**	Patients complained about poor staff attitude.	Patient satisfaction survey (B7, B9, B11)	Educate staff on the importance of having a positive attitude towards patients- *staff-patient relationship* (B7, B9, B11)
**16**	Poor level of cleanliness within the facility. Water-logged toilets that were not regularly cleaned. Poor patient satisfaction due to poor state of the ward and environment. Untidy service areas because some housekeeping staff were not performing assigned duties. Patient wards were not conducive for habitation because of mosquitoes	Patient satisfaction survey (B15, B17)<br>Patient complaint (C20)	Get management buy-in and advocate to them to address plumbing issues- *clean and conducive environment* (B15)
			Develop cleaning roster and supervisors should monitor compliance of cleaners- *clean and conducive environment* (B15) (C11).
			Constitute a committee to look into cleanliness of hospital environment - *clean and conducive environment* (B17)
			Renovate wards and clinics- *clean and conducive environment* (B17) (C17, C20)
			Caution erring housekeeping staff- *clean and conducive environment* (C20)
			A copy of housekeeper's job allocation and phone numbers should be pasted at each nursing station to know erring housekeepers- *clean and conducive environment* (C20).
			Train cleaners - *clean and conducive environment* (B8)
			Install electronic insect killer in the wards/periodic use of insectides in the ward - *clean and conducive environment* (C20)
**17**	Patients complained about the quality of food served. Plausible reasons included inadequate training of cools, no functional diet committee, non-availability of some equipment, lack of variety of meals, patients on special diets not enlightened	Patient complaint (B8)<br>Staff observation (B8)	Train all cook- *quality meals* (B8)
			Place catering officers on weekend calls - *quality meals* (B6)
			Mandate catering officers to fill an attendance sheet to track compliance with weekend calls- *quality meals* (B6).
			Refurbish the kitchen and its call room- *quality meals* (B6).
			Constitute a diet committee- *quality meals* (B6)
			Provide the requisite kitchen equipment, ingredients and utensils- *quality meals* (B6, B8).
			Develop and post a food menu that reflects variety- *quality meals* (B8)
			Enlighten patients on special diets (e.g., diabetics) that their meal may taste different- *quality meals* (B6).
			Assign someone to taste the food before it is served- *quality meals* (B8)

5. Foundations- governance: 1 problem identified and 1 change idea tested

Under this domain, the number of facilities documented to have identified problems and developed change concepts and ideas during the study period:

- 0 of 6 PHCs
- 1 of 14 public hospitals (labelled B15)
- 0 of 8 private facilities

S/N	Problem description	Method of problem identification	Change ideas –*change concepts*
1	Need to get management's political commitment for the selected change ideas	1. Recommendation by Safe care initiative (B15)	QI reports, including change ideas are to be reviewed at Management meetings- *buy-in of the management* (B15).

Olaniran AA, *et al. BMJ Open Qual* 2022; 11:e001532. doi: 10.1136/bmjoq-2021-001532

6. Foundations- workforce: 8 problems identified and 11 change ideas tested

Under this domain, the number of facilities documented to have identified problems and developed change concepts and ideas during the study period:

- 1 of 6 PHCs (labelled A2)
- 2 of 14 public hospitals (labelled B12, B18)
- 3 of 8 private facilities (labelled C15, C20, C25)

S/N	Problem description	Method of problem identification	Change ideas-*change concepts*
**1**	Non-QI staff members were demotivated from the additional documentation and activities required by QI as it was considered a major contributor to workload.	Not documented	Institute monetary awards to best junior and senior staff that have been supportive of QI activities as well as compliance with standards of practice in the facility- *staff welfare* (C25).
**2**	Shortage of doctors in the facility as doctors often reject posting to the facility because of its remote location and poor state of the roads leading to the area.	Not documented	Renovate staff quarters to accommodate newly posted doctors to forestall the need to travel to work- *staff welfare* (B12).
**3**	To ensure continued commitment of QI team members.	Not documented	Giving of incentive to all QI members- *staff welfare* (B18)
**4**	Complaints that staff should have rights	Not documented	Staff rights should be written alongside patient rights- *staff welfare* (C20).
			Develop a platform such as suggestion box where staff members can provide suggestions or complain - *staff welfare* (C20).
**5**	Lack of cordial relationship among staff with frequent disagreement between staff members.	Not documented	Create a WhatsApp group for staff members to interact- *staff welfare* (C15).
**6**	Staff lateness was contributing to delay in attending to patients	Not documented	Provide staff attendance registers in all units to track staff punctuality- *staff discipline* (A2) (C15).
			Institute staff disciplinary committee to review staff conduct and make recommendations- *staff discipline* (C20)

**7**	Problem with proper handing over of shift duty with instructions not being passed on from one shift to the other.	Not documented	Handing over of shift duty should be documented- *staff discipline* (C25).<br>Ensure hand over of communication materials e.g. mobile phone- *staff discipline* (C15)
**8**	Some staff were not wearing decent attires with others refusing to wear ward coats.	Not documented	Images of decent dressing should be shared among staff and memo reiterating the importance to be shared to all departments- *staff discipline* (C20)
			Staff refusing to wear ward/lab coats should be identified by designated staff members with the aim of calling them to order- *staff discipline* (C20)

Olaniran AA, *et al. BMJ Open Qual* 2022; 11:e001532. doi: 10.1136/bmjoq-2021-001532

7. Foundations- tools: 10 problems identified and 19 change ideas tested

Under this domain, the number of facilities documented to have identified problems and developed change concepts and ideas during the study period:

- 4 of 6 PHCs (labelled A1, A2, A3, A5)
- 6 of 14 public hospitals (labelled B3, B6, B7, B10, B12, B18)
- 2 of 8 private facilities (labelled C15, C20)

S/N	Problem description	Method of problem identification	Change ideas-*change concepts*
1	Unavailability of fresh blood in the blood bank including O+ve blood (universally accepted blood group) due to government policy that blood donation should be voluntary.	Facility register (B3, B10)	Sensitise facility staff & host community members (including religious houses) on voluntary blood donation- *availability of commodities* (B3, B7, B10).
			Provide donor incentive e.g. blood tonic - *availability of commodities* (B3, B7, B10)
			Print and disseminate flyers & posters on the importance of blood donation- *availability of commodities* (B3, B7).
			Collect blood from other facilities - *availability of commodities* (B3).
			Establish a blood transfusion committee to oversee blood transfusion in facility- *availability of commodities* (B3, B7, B12).
			Generate a directory of voluntary donors, especially those that are O positive - *availability of commodities* (B3).
2	Poor availability of supplies required for the management (adherence to protocol) of severe pre-eclampsia/eclampsia	Not documented	Prepack and supply drugs for eclampsia management ("Eclampsia pack") - *availability of commodities* (B18)
3	Stockout of drugs at the pharmacy because of inefficiencies of the new pharmacy staff	Not documented	Inventory taking should be done by a staff with requisite skills in stock taking- *availability of commodities* (C20).
			Prompt reordering of drugs to prevent stockout - *availability of commodities* (C20).
4	The need for good Ambu bag as the available does not fit well with the oxygen connector.	Not documented	Get a good Ambu bag and a connector to attach it to oxygen supply - *availability of equipment* (C15).

**5**	The need to promptly identify faulty equipment and repair accordingly as it was contributing to waiting time	Not documented	Send a memo to inform staff to document faulty equipment in their respective departments - *availability of equipment* (C20)<br>Employ a facility manager to facilitate fixing/replacement of faulty equipment identified in the various departments- *availability of equipment* (C20)<br>Meet with head of engineering to repair faulty equipment and install the new machine- *availability of equipment* (B6).
**6**	Inadequate number of vital sign equipment often means that nurses have to take turns to use the available equipment, consequently increasing waiting time	Not documented	Purchase more BP apparatus, weighing scale and thermometers - *availability of equipment* (A1)
**7**	Non-availability of wash hand basin with taps, no disposable hand towels to dry hands after washing to prevent the spread of infection	Not documented	Purchase more Veronica buckets with stands and bowls for handwashing - *availability of equipment* (A2)
**8**	Non-availability of running water during a power cut when the water pumping machine cannot be powered	Not documented	Purchase a storage tank to store pumped water such that water is available for longer- *availability of utility* (A3)
**9**	Inadequate power supply affecting service delivery	Not documented	Purchase a generator to power service units - *availability of utility* (A5)<br>Purchase and use machines that don't rely on electricity- *availability of equipment* (A2).
**10**	Inadequate power supply making it difficult to power laboratory equipment.	Not documented	Connect all lab equipment to facility generator- *availability of utility* (A5).

Olaniran AA, *et al. BMJ Open Qual* 2022; 11:e001532. doi: 10.1136/bmjoq-2021-001532
